# Supplementary material for: High fidelity sensory-evoked responses in neocortex after intravenous injection of genetically encoded calcium sensors
Source: Front Neurosci. 2023 May 12;17:1181828. doi: 10.3389/fnins.2023.1181828 (PMC10213453; doi:10.3389/fnins.2023.1181828)
Supplement: Supplementary file 3 [file Data_Sheet_1.PDF]

Supplementary Material

High fidelity sensory-evoked responses in neocortex after intravenous injection of genetically encoded calcium sensors

Austin Leikvoll and Prakash Kara*,

*** Correspondence:** pkara@umn.edu

The supplementary material contains the following:
1. One Supplementary note
2. Three Supplementary figures
3. Two Supplementary videos

1 Supplementary note

Viral titer selection. The Gradinaru lab has provided a guidebook on the use of blood-brain-barrier-crossing AAV capsids (see Challis et al. 2019 Nature Protocols). They recommend that users begin by injecting 1×10^{11} viral genomes per mouse. From this starting point, the user should empirically evaluate whether transgene expression levels meet the user's needs. If 1×10^{11} viral genomes/mouse does not give sufficient labeling for a user's purposes, the viral titer can be increased until labeling is sufficient. Challis and colleagues state that they typically use between 1×10^{11} and 5×10^{11} viral genomes. Thus, in our pilot testing of this method, we injected 1×10^{11} viral genomes of AAV-PHP.eB per mouse. However, when performing *in vivo* two-photon imaging of GCaMP-labeled neurons with this lower titer, 80–100 mW of power was needed in cortical layer 2/3 when using the high NA Olympus objective. Thereafter, we increased our injections to 4×10^{11} viral genomes per mouse. This was a sufficient titer for two-photon imaging with reasonable laser powers (<30 mW in layer 2/3 with the high NA Olympus objective) and thus all data presented in this study was based on injecting 4×10^{11} viral genomes per mouse, as specified in the "Materials and methods" section.

2 Supplementary figures

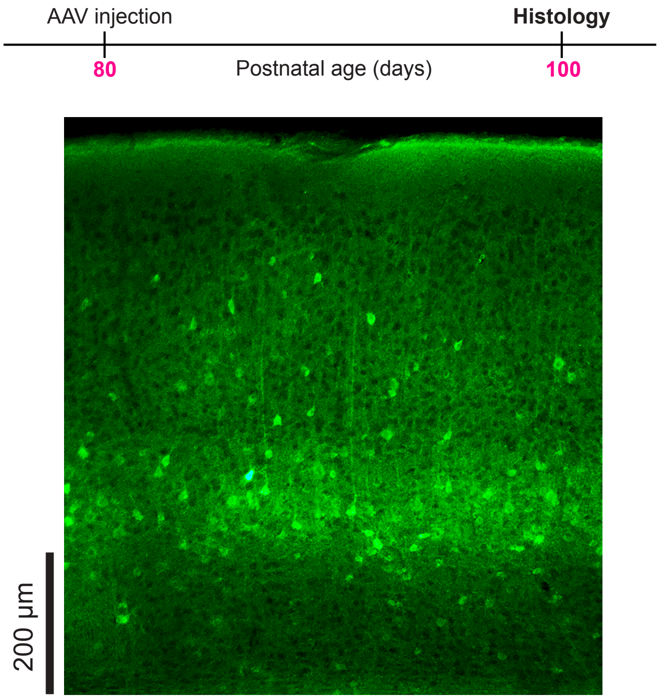

Supplementary Figure 1. Histological coronal section that was imaged 20 days after intravenous injection of AAV-PHP.eB. The mouse age at AAV injection was 80 days postnatal.

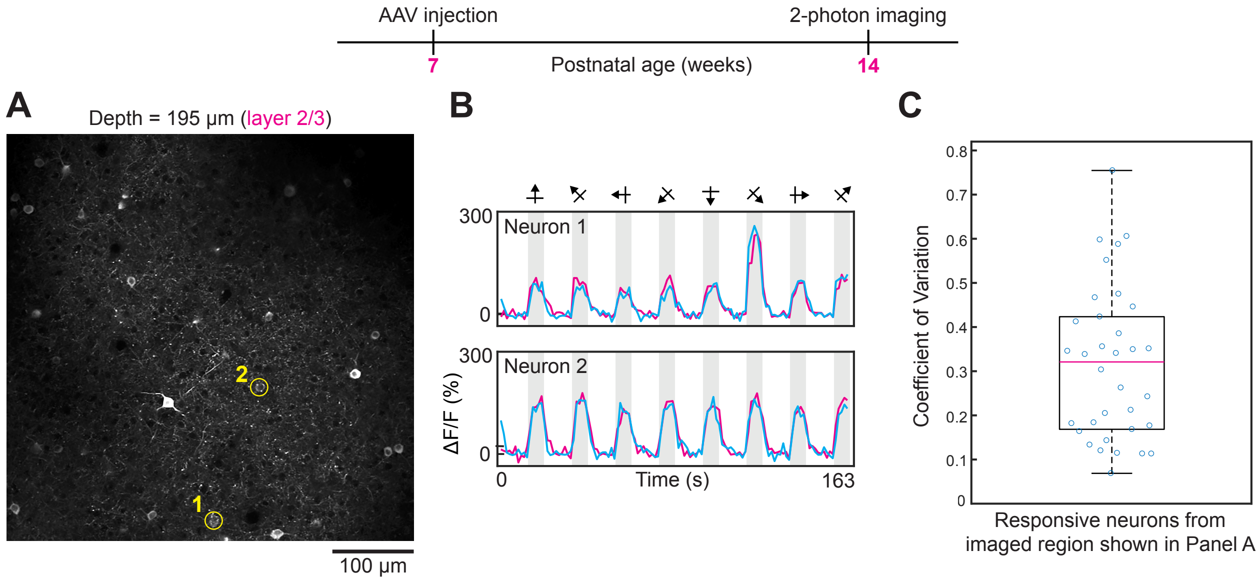

Supplementary Figure 2. Two-photon imaging of neural responses 7 weeks after intravenous injection of AAV GCaMP7. (**A**–**B**) Data from layer 2/3 (imaging plane 195 μm below the pia). The average laser power used was 27 mW. The two circled neurons in panel A (numbered 1–2) were selected to show tuned and untuned visual responses, respectively (see time courses in panel B). For all time courses shown in this figure, two trials are overlaid. (**C**). Box plot of the coefficient of variation of calcium responses for neurons that were significantly responsive to visual stimuli ($p <$ 0.01, ANOVA across baseline and eight directions over two trials).

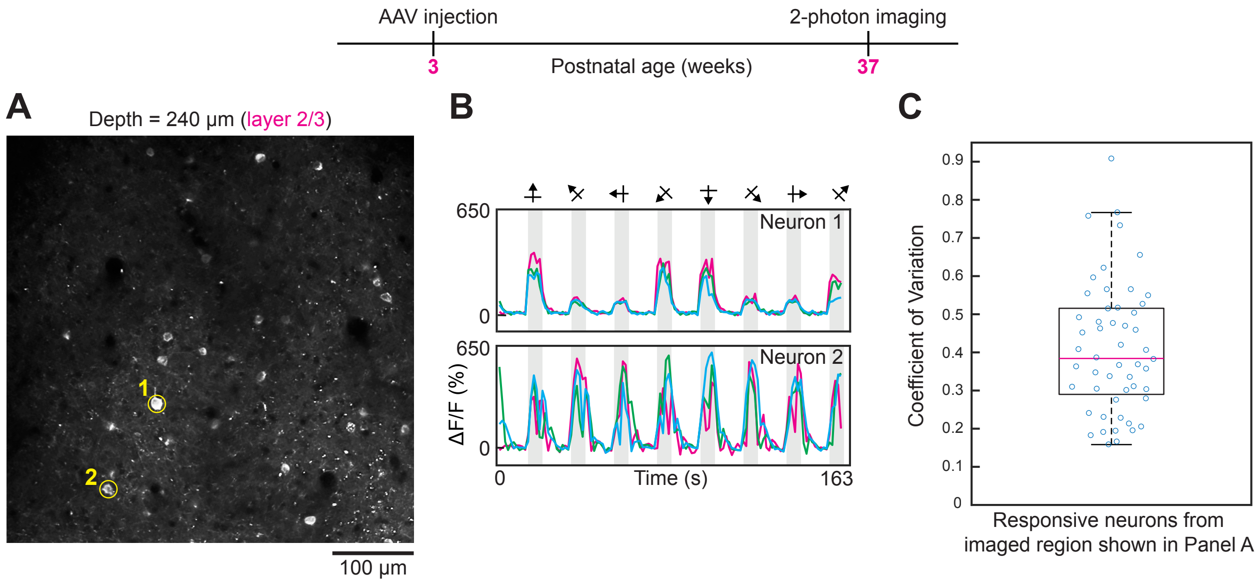

Supplementary Figure 3. Two-photon imaging of neural responses 34 weeks after intravenous injection of AAV GCaMP7. (**A**–**B**) Data from layer 2/3 (imaging plane 240 μm below the pia). The average laser power used was 45 mW. The two circled neurons in panel A (numbered 1–2) were selected to show tuned and untuned visual responses, respectively (see time courses in panel B). For all time courses shown, three trials are overlaid. (**C**). Box plot of the coefficient of variation of calcium responses for neurons that were significantly responsive to visual stimuli ($p < 0.01$, ANOVA across baseline and eight directions over three trials).

3 Supplementary videos legends

Supplementary video 1. *In vivo* cortical volume demonstrating cortical expression across layers following intravenous injection of AAV GCaMP7. This video is a rotating side perspective of the static 3D volume reconstruction shown in Figure 1B.

Supplementary video 2. Raw two-photon imaging frames showing GCaMP7 responses in layer 2/3 of mouse V1 *in vivo*. Raw data frames (left) and frame-locked presentation (right) of blank (gray) and drifting grating visual stimuli. Data are shown for 25 imaging frames: 10 frames blank, followed by 5 frames of visual stimulation and then 10 frames blank. The duration of each frame was 1.14 s. The entire x-y field of view shown in the 2 two-photon imaging frames represents a region of 500 µm × 500 µm. Movie corresponds to data shown in Fig. 4D–F (16 weeks after intravenous injection).
